# Changing Degree of Convective Organization as a Mechanism for Dynamic Changes in Extreme Precipitation

**DOI:** 10.1007/s40641-020-00157-9

**Published:** 2020-05-04

**Authors:** Angeline G. Pendergrass

**Affiliations:** 1 Climate and Global Dynamics Laboratory, National Center for Atmospheric Research, PO Box 3000, Boulder, CO 80303 USA; 2 Institute for Atmospheric and Climate Science, ETH Zurich, Zurich, Switzerland

**Keywords:** Extreme precipitation, Convective organization, Climate change

## Abstract

**Purpose of Review:**

What does recent work say about how changes in convective organization could lead to changes in extreme precipitation?

**Recent Findings:**

Changing convective organization is one mechanism that could explain variation in extreme precipitation increase through dynamics. In models, the effects of convective self-aggregation on extreme precipitation are sensitive to parameterization, among other factors. In both models and observations, whether or not convective organization influences extreme precipitation is sensitive to the time and space scales analyzed, affecting extreme precipitation on some scales but not others. While trends in observations in convective organization associated with mean precipitation have been identified, it has not yet been established whether these trends are robust or relevant for events associated with extreme precipitation.

**Summary:**

Recent work has documented a somewhat view of how changes in convective organization could affect extreme precipitation with warming, and it remains unclear whether or not they do.

## Introduction

Extreme precipitation events are often associated with organized convection [1, 2•], but theory for how extreme precipitation responds to global warming is based largely on arguments assuming circulation associated with extreme events does not change with warming [3, 4]. A model whose purpose is to simulate how extreme precipitation changes with global warming should span scales from convection through global energy and water balance; however, the models that are available at present can generally do one or the other, not both. This makes it difficult to assess how circulation could change with warming in ways that are relevant to extreme precipitation. What would it mean for extreme precipitation if convective organization were to change with warming? This review explores recent research related to this question.

There is a lot we have known for some time about how extreme precipitation responds to anthropogenically forced warming from theory, models, and even observations. Evidence from theory and model projections has pointed to increasing extreme precipitation with warming over most of the planet. Observed changes in precipitation that are broadly consistent with model projections at the hemispheric scale have been detected and attributed to anthropogenically-forced warming [5].

In the absence of changes in circulation with warming, the response of extreme precipitation would increase due to thermodynamic factors. Relative humidity is expected to change little with warming (e.g., [6–8]); thus, absolute humidity increases with temperature. If extreme precipitation intensity roughly follows moisture convergence in extreme events, then in the absence of changes in circulation, the intensity of extreme precipitation would increase with moisture. But over the last decade or so, arguments have been made based on observations that increases in extreme precipitation could be much larger than the thermodynamic scaling implies [9]. Some climate model projections also indicate possible increases in extreme precipitation with warming that could be larger than the increase in moisture, though with large variation among models, particularly in the tropics [10, 11].

In addition to the thermodynamically driven increases in extreme precipitation, changes in circulation—dynamics—can drive changes in extreme precipitation that deviate from the thermodynamic scaling [12]. Large-scale shifts in circulation could also drive dynamic changes that could influence extreme precipitation change, like the strength of the Walker cell or changes in location of storm tracks, and these factors are relatively well studied [13], though the effect of shifts in precipitating features on extremes precipitation will depend on whether extremes are defined with respect to a local baseline or a broader one, which will be discussed later. Dynamic scaling is important for capturing the spatial pattern of precipitation change projected by climate models [14]. The inter-model spread in extreme precipitation change is driven by dynamic, rather than thermodynamic factors [10]. Overall, most of the remaining uncertainty in the future extreme precipitation originates from the dynamics, which are associated with a variety of scales, from global to storm scale.

Convective organization is another factor that could influence the dynamic changes of extreme precipitation. Convective organization is a broad term encompassing a variety of phenomena and can perhaps most easily be understood by its contrast, which is random (or popcorn) convection—convective cells that are distributed randomly in space and time. Convective aggregation is when convective cells clump, or cluster together, in space, and are surrounded by comparatively dry regions. Convective self-aggregation is a phenomenon that occurs when the energy and momentum fluxes associated with convective cells and the dry regions in which they are embedded in cause the convective cells to move together; at least one but possibly more mechanisms can cause self-aggregation [15, 16]; a body of work studying it has developed [17]. In addition to aggregation, convective organization also encompasses phenomena including mesoscale convective systems (MCSs) [18, 19], tropical cyclones, and extra-tropical cyclones.

Organized convection, for which small-scale processes play an important role, is not captured at the scales resolved by typical climate models at present. This means more creative and less direct approaches to studying its effects are called for. For example, high-resolution regional model simulations have been used to examine the response of extreme precipitation to warming in one particular type of organized state—squall lines—without changes in the degree of organization and showed that the response of extreme precipitation does differ slightly in organized convection compared with unorganized convection [20, 21].

In the last 5 years, changes in organization of convection have emerged as a potential mechanism that could drive changes in extreme precipitation with warming. The key question addressed in this review is: what have we learned from recent work about whether and how changing convective organization could affect extreme precipitation?

## Changing Convective Organization as a Mechanism for Extreme Precipitation Change

Even though convection is parameterized in climate models, these models can still exhibit convective self-aggregation. In global radiative-convective equilibrium (RCE) simulations that have been run with a few climate models, convective aggregation varies with sea surface temperature (SST) [22–24]. Each of these sets of simulations is carried out with a model with the physics of a climate model and either a fully global or near-global domain, in contrast to small domain but convection resolving simulations that may be far from RCE [25]. These simulations have no land surface (only prescribed SST), no rotation, and a single globally fixed SST. Over some SST ranges, convection remains disorganized and randomly distributed throughout the domain. But above or even below an SST threshold, convection aggregates, though the specific thresholds where this occurs vary among the models. Changes between aggregated and disorganized convection with increasing SST substantially alter the atmospheric temperature, relative humidity, and circulation throughout the domain—the degree of aggregation affects the large-scale circulation and tropospheric state. This includes the extreme daily precipitation, whose intensity increases, in one case well beyond thermodynamic scaling, illustrating that convective aggregation can exert large influence on extreme precipitation by altering atmospheric circulation associated with extreme events [24]. In this set of simulations, the upward vertical velocity increases as convective aggregation increases, which drives more condensation, and is also consistent with increased moisture convergence. While these simulations are idealized, these types of simulations resemble the tropical atmosphere and its response to warming [23, 26]. In an analogous configuration that includes background large-scale circulation (unlike the illustrative simulations discussed above), changing convective organization also affects extreme daily precipitation [27•].

In summary, in climate model simulations, changes in convective organization can drive changes in extreme precipitation dynamically, by altering the circulation associated with extreme events. Though these studies have all used simulations that were idealized to varying degrees, they are potentially relevant to Earth’s tropics. And despite that these all use conventional climate models, the behavior of their convective aggregation is qualitatively similar to cloud resolving model simulations (e.g., [16]). Next, we will discuss some limitations to the relationship between convective organization and extreme precipitation that have been identified in recent work.

## Model and Parameterization Dependencies

The simulations discussed in the previous section all included parameterized convection. The choice of parameterization has a large effect on convective organization and also how it affects extreme daily precipitation [27•]. One challenge for simulating the role of organized convection in climate change is the wide range of scales that should be resolved to capture all the important processes. Ideally, a simulation would resolve scales as small as convection and also as large as the global water and energy budgets, but representing all of these scales simultaneously is not currently feasible. Coarse-resolution climate models resolve global scales and parameterize convection. Currently, global convective-permitting models are too expensive to carry out the length and number of simulations to study extreme events. Instead, most studies either use climate models that parameterize convection or regional models that resolve convection to varying degrees but do not explicitly represent global water and energy balance.

One approach that makes a different compromise is superparameterization [28]. These use coarse-resolution global models, but within each grid cell is embedded a 2D simulation of convection. By collapsing the calculation to two dimensions, higher resolution can be achieved, so that convection can be resolved. CAM is one conventional climate model atmosphere component that has a moderately large dynamic increase of tropical extreme daily precipitation in response to warming [29]; replacing its convective parameterization with superparameterization changes its extreme precipitation response [30]. This has been interpreted as implying that improving the representation of convection, particularly its dependence on parameterization, can remove the large and percentile-dependent component of the extreme precipitation response, bringing it back toward convergence to thermodynamic scaling with increasing extremes [31•]. While conventional and superparameterized CAM have substantial differences in their present-day extreme precipitation [32], observational uncertainty precludes confidence about which is better [33].

However, the theory for convergence of extreme precipitation change to thermodynamic scaling does not hold when convective organization changes with warming. For convection whose degree of organization does not change, the circulation (specifically, vertical velocity) scales with CAPE, and CAPE scales with Clausius-Clapeyron [34, 35], though this does not necessarily extend to large-scale ascent for a climate model grid box [36]. Because convective organization alters temperature and humidity throughout the troposphere, changes in convective organization with warming would also be expected to affect the response of CAPE to warming. While superparameterized models can, perhaps arguably, simulate organized convection, including phenomena like the MJO that are not typically captured by models with conventional parameterizations [37], the thermodynamic scaling of extreme precipitation in the superparameterized CAM simulations indicates that convective organization might not change with warming in this experiment with this model.

One indication of the importance of parameterizations in models is the tradeoff between increases in extreme and non-extreme precipitation, which explains some of the inter-model spread in extreme precipitation increase with warming [38]. In climate models with larger increases in extreme precipitation, non-extreme precipitation compensates by increasing less or even decreasing; meanwhile, these models also have larger increases in global-mean precipitation. In aggregated states compared with disorganized ones, a larger fraction of total precipitation falls as extreme precipitation [24], which implies that the tradeoff between extreme and non-extreme precipitation is consistent with large increases in convective organization in models with larger increases in extreme precipitation. This piece of evidence would be consistent with a correlation between convective organization and extreme precipitation change in climate models.

In summary, convective organization and its effects on extreme precipitation are sensitive to convective parameterizations and configurations in model simulations.

## Mismatch in Responses of Extreme Precipitation Across Time and Space Scales

The behavior of extreme precipitation depends on how it is defined, in terms of the time and space scale. Three studies have revealed different aspects of how extreme precipitation depends on convective organization. They show that it differs from coarser to finer spatial averaging scale (order 100 compared with 1 km) and longer to shorter timescales of precipitation (daily accumulation to instantaneous rate).

While we have seen that daily precipitation accumulation depends on convective organization in model simulations, instantaneous precipitation does not always differ between organized and disorganized precipitation. Tradeoffs between changes in updraft speed and buoyancy due to graupel offset each other to result in similar instantaneous precipitation rates between organized and disorganized states. But because events are more persistent in aggregated states, daily precipitation accumulation is higher, which is offset by more grid points that are dry throughout the day [39•]. Domain-total precipitation varies in a complementary way in space—because precipitation rates are similar between organized and unorganized precipitation, domain-total precipitation depends mostly on the precipitating area fraction, rather than the degree of organization [40].

The behavior of extreme precipitation can vary across spatial scales, complementary to its behavior across timescales. Changing model resolution changes the balance between convective and large-scale parameterized precipitation and can also affect the magnitude of extreme precipitation [41, 42]. In the superparameterized CAM experiments described above, at coarse grid box scale, the precipitating area increases for more extreme events. In contrast, at sub-grid box scales, precipitating area decreases slightly for more extreme events [31•]. This indicates that the number and/or size of events are the primary control of extremes at coarse spatial scales, while their intensity is the main control at smaller spatial scales. Furthermore, sub-grid scale extreme events do not necessarily coincide with grid scale extreme events.

Observational analyses are also consistent with the notion that short-timescale intensity is similar between organized and disorganized states [2•]. Convective organization can be quantified in various ways. One method is based on classifying different cloud regimes. Some regimes are recognized as associated with organized convection, others with isolated, unorganized convection, among other weather features. Separating organized and disorganized convection based on cloud regime classification, the intensity of precipitation at points with precipitation is similar, suggesting that differences in precipitation rate averaged over the entire regime are due to the fraction of area in which precipitation occurs, rather than the intensity of the precipitation [2•].

A different observational approach focusing on convective aggregation is to classify the degree of aggregation using an index based on convective cores, identified in brightness temperature or vertical velocity, and a measure of how clustered they are in space [43, 44]. Applying this approach to scenes collocated with satellite precipitation estimates shows that local extreme precipitation depends on the degree of aggregation, while extremes of domain-averaged precipitation does not [45•]. This finding differs from other observational results in that the finer spatial scales are sensitive to organization while coarser scales are not [2•, 39•]. Meanwhile, other studies using a similar classification of convective aggregation find that the vertical distribution of clouds [46], humidity, and precipitation efficiency are quite different for different degrees of convective aggregation [43].

Finally, extremes can be defined in many different ways, and these differences in definition can have quantitative and qualitative effects on their interpretation. One aspect of the definition is whether extremes are defined locally or over an entire domain. Say, for example, the ITCZ were to change by only shifting in latitude. If we define extreme relative to a baseline that includes the entire tropical domain, and if we are only interested in the properties of extremes over the domain as a whole, then a shift of the ITCZ in latitude will have no effect. This is typically the type of analysis that is done with RCE simulations, where we know that the individual locations are not important; all locations in the domain (which is often periodic) are the same. On the other hand, if we define extreme precipitation relative to a baseline that is calculated separately at each grid point and examine the change in extreme precipitation at each grid point, then we will find large changes in extreme precipitation in response to the shift in space of the ITCZ. The local definition would matter more for considering local impacts of extremes, so for some purposes, this is an appropriate way to define extremes, even though it can lead to different interpretations. The absolute response can also depend on how the domain is defined. Another example of differing definitions of extremes leading to different conclusions is whether dry times or all times are used to calculate percentile thresholds. If the total dry frequency changes from the base state to the changed state, then changes defined relative to all times and dry times will diverge, and this can also lead to qualitatively different conclusions about extreme precipitation change [47].

In summary, multiple studies using different models and observational datasets agree that extreme precipitation differs between fine and coarse scales in space or time. They agree that not only does the behavior of extremes depend how rare an event is (i.e., what percentile of the distribution it represents), but also the temporal and spatial scale considered. Studies that diagnose the dependence of extreme precipitation for organized and unorganized states on multiple spatial scales disagree; however, about whether larger- or smaller-scale extremes vary with convective organization: two studies show that fine scales (in space or time) are similar between organized and disorganized states, but organization increases persistence or precipitating area, and thus, dependence on the degree of organization emerges for coarser-scale extremes. In contrast, the most recent observational study shows that local extremes depend on organization, while extremes of domain-averaged precipitation (given the same number of embedded convective cells) do not [45•]. Furthermore, temporal and spatial scales are connected; spatially organized states are often more persistent in time, while disorganized states have more intermittent precipitation. Finally, the differences among metrics that are used to define and quantify convective organization may well contribute to the discrepancies among studies.

## Trends in Convective Organization with Warming

Simulations support the notion that on some scales and for some configurations, changes in convective organization could influence extreme precipitation change, and observations show some indication that convective organization could influence extreme precipitation. But, are there observable trends in convective organization or associated extreme precipitation? During recent decades, organized convective cloud regimes identified in satellite observations of the tropics increased in frequency [48] at the expense of unorganized regimes; the spatial pattern of these changes is closely related to the spatial pattern of the change in mean precipitation [49]. While ref. [49] showed the effect of changes in convective organization on mean precipitation, an association between trends in organized convection and trends in extreme precipitation in observations has not been documented.

Climate model simulations might be able to provide insight about responses to anthropogenic forcing and also what the role of natural variability could be. Some, but not all, relevant studies show that convective aggregation could be favored by warmer surface temperatures, indicating that convective organization could increase in response to warming [15, 16, 50]. The large increases, and lack of large decreases, in extreme precipitation with warming in climate model projections are consistent with increasing, but not decreasing, convective organization with warming. Though, as we have seen, changes in the degree of convective aggregation are quite sensitive; they depend on resolution, parameterizations, and the model considered. Convective organization is one aspect of the response to climate change that is, at best, poorly represented in climate models and could potentially be (though has not yet been) parameterized in coarse-resolution climate models [51, 52]. In addition to extreme precipitation changes in convective organization have even been discussed as potentially affecting mean precipitation and climate sensitivity [53].

## Discussion

There are a variety of phenomena involving organized convection that could influence extreme precipitation. We have focused mostly on convective aggregation. But mesoscale and synoptic dynamics also drive phenomena like MCSs, and tropical and extra-tropical cyclones, within which organized convection can be embedded; these dynamics are also driven by convection through its diabatic heating. The convective systems are only beginning to be resolved by global climate models [54], despite that they are an important part of the climate response to global warming. A large part of the response of precipitation associated with tropical cyclones and extratropical cyclones can potentially be explained by thermodynamic responses [55–58]. Each phenomenon involving organized convection should be considered on its own as well as holistically for its role in the global hydrologic cycle. And, it should be noted that, perhaps counterintuitively, extreme precipitation events are not necessarily associated with extreme convection [59].

One study has shown that replacing the conventional parameterization in CAM with a superparameterization removed the divergence from thermodynamic scaling [31•]. It remains unclear how general of a feature of conventional parameterization the divergence of extreme precipitation from thermodynamic scaling is. Not all conventionally parameterized climate models have an increase in extreme precipitation that is substantially larger than thermodynamic scaling, so a large dynamic increase is not an entirely general feature of parameterized convection [11]. One test for how robustly superparameterization removes the divergence from thermodynamic scaling would be to examine the state dependence of the extreme precipitation response in superparameterized CAM. It is possible that conventionally parameterized CAM transitions toward increasing convective organization, while superparameterized CAM does not, only because its SST threshold is higher or lower than conventional CAM. A second test would be to replace conventional parameterizations with superparameterizations in other climate models with a large divergence from thermodynamic scaling (such as GFDL-ESM 2 or IPSL-CM5A-LR). If replacing conventional parameterization with superparameterization removed the divergence from thermodynamic scaling in these other models as well as CAM, it could indicate that the divergence from thermodynamic scaling is a numerical artifact in climate models which do not resolve convective scales. But if the effect did not persist in other models and states, this might be because of the nonlinear dependence of self-aggregation on temperature and parameterization. Meanwhile, even if replacing conventional with superparameterizations consistently removes the divergence from thermodynamic scaling, this still may not accurately reflect changes in convective organization (or lack thereof), because superparameterized simulations still have a scale separation between the grid box and embedded cloud resolving scale that lies roughly at the size of organized convective systems [36].

Finally, while we have largely tried to focus on the potential influence that changes in dynamics due to changing convective organization could have on extreme precipitation, microphysics may play also play an important role. Compensation between microphysics and dynamics has been identified in observations [60] and with changes in organization in model simulations [39•]. Simulated scaling of extreme precipitation with warming can vary with how microphysics is represented [61]. Microphysics remains a key uncertainty in understanding the connection between convective organization and extreme precipitation [62]. The representation of turbulence can also influence convective organization [44], so this related aspect of small-scale physics is yet another dimension that requires attention.

## Conclusions

Changing convective organization has been put forward in the last 5 years as one mechanism that could drive dynamic changes in extreme precipitation, beyond those of the thermodynamic scaling. Increase of extreme precipitation with increasing convective organization has been identified in models, on some temporal and spatial scales, lending support to the potential relevance of the mechanism. We have learned that in observations, as in models, there is some evidence that aspects of extreme precipitation depend on convective organization.

We have also learned that the effects of convective organization on extreme precipitation have complexities. The dependence of extreme precipitation on convective organization varies with how precipitation is represented. The effects of organization also vary with temporal and spatial scale.

Unanswered questions remain. Observations of mean precipitation have shown that convective organization might be increasing. Has this trend persisted in the intervening decade of data that is now available? And, does the trend toward increasing convective organization extend to the subset of events that are associated with extreme precipitation? These questions could be investigated with new observations, as the duration of satellite measurements lengthens, and should be evaluated with multiple datasets, since estimates of extreme precipitation have important differences among datasets [33].

Is the uncertainty in the response of extreme precipitation to global warming across climate model projections driven by changing convective organization? Some climate models with parameterized convection from the previous generation (CMIP5) have extreme precipitation that converges to the thermodynamic scaling, which could be consistent with no change in the degree of convective organization with warming. Meanwhile, others had large increases in the most extreme tropical precipitation events, beyond thermodynamic scaling, potentially consistent with increasing convective organization. The tradeoff between extreme and non-extreme precipitation change across models would be consistent with a correlation between changing convective organization and extreme precipitation change, but this needs further investigation.

A new generation of climate model simulations is becoming available (CMIP6) [63], with more models, and some that include updated convective parameterizations and additional processes. Does the large uncertainty across models persist in this new and hopefully improved generation of simulations? Does it persist in the relatively high-resolution subset of experiments in CMIP6 [64]? And if so, do changes in convective organization explain an important component of that variation? Whether or not it persists, since climate models still do not represent convective scales, what does this mean for the real world?

We will continue to see advances in modeling. One advance that could be important for this particular topic would be a parameterization for organized convection [51, 65, 66]. It seems possible that a modeling approach tailored specifically to the hydrologic cycle, including precipitation and convective organization in a realistic, global, and coupled modeling framework, could improve on current approaches for addressing these questions. Variable-resolution grids provide simulations on the global scale while also allowing finer resolution in limited regions, which are a potential improvement on limited area models which have no representation of the global energy and water budgets [67]. Perhaps the continuing advances in machine learning could be harnessed into a new type of convective parameterization that accounts for organized convection [68] which could be implemented in conventional climate models. And, advances in computing will hopefully bring us ever closer to, and eventually enable us to carry out, global coupled convective-permitting simulations.
